# Top-Down Characterization of an Antimicrobial Sanitizer, Leading From Quenchers of Efficacy to Mode of Action

**DOI:** 10.3389/fmicb.2020.575157

**Published:** 2020-09-25

**Authors:** Franziska Wohlgemuth, Rachel L. Gomes, Ian Singleton, Frankie J. Rawson, Simon V. Avery

**Affiliations:** ^1^School of Life Sciences, University of Nottingham, Nottingham, United Kingdom; ^2^Faculty of Engineering, University of Nottingham, Nottingham, United Kingdom; ^3^School of Applied Sciences, Edinburgh Napier University, Edinburgh, United Kingdom; ^4^School of Pharmacy, University of Nottingham, Nottingham, United Kingdom

**Keywords:** antimicrobial sanitizer, mode of action, oxidative stress, methionine, fungi, yeast, soil organics, electrolyzed water

## Abstract

We developed a top-down strategy to characterize an antimicrobial, oxidizing sanitizer, which has diverse proposed applications including surface-sanitization of fresh foods, and with benefits for water resilience. The strategy involved finding quenchers of antimicrobial activity then antimicrobial mode of action, by identifying key chemical reaction partners starting from complex matrices, narrowing down reactivity to specific organic molecules within cells. The sanitizer electrolyzed-water (EW) retained partial fungicidal activity against the food-spoilage fungus *Aspergillus niger* at high levels of added soils (30–750 mg mL^–1^), commonly associated with harvested produce. Soil with high organic load (98 mg g^–1^) gave stronger EW inactivation. Marked inactivation by a complex organics mix (YEPD medium) was linked to its protein-rich components. Addition of pure proteins or amino acids (≤1 mg mL^–1^) fully suppressed EW activity. Mechanism was interrogated further with the yeast model, corroborating marked suppression of EW action by the amino acid methionine. Pre-culture with methionine increased resistance to EW, sodium hypochlorite, or chlorine-free ozonated water. Overexpression of methionine sulfoxide reductases (which reduce oxidized methionine) protected against EW. Fluoroprobe-based analyses indicated that methionine and cysteine inactivate free chlorine species in EW. Intracellular methionine oxidation can disturb cellular FeS-clusters and we showed that EW treatment impairs FeS-enzyme activity. The study establishes the value of a top-down approach for multi-level characterization of sanitizer efficacy and action. The results reveal proteins and amino acids as key quenchers of EW activity and, among the amino acids, the importance of methionine oxidation and FeS-cluster damage for antimicrobial mode-of-action.

## Introduction

Chemical sanitizers and disinfectants have applications for control of microbial contamination and growth in diverse settings, including healthcare and food industries as well as domestic use, with a global market approaching USD 20 billion. That looks set to increase markedly with the heightened public awareness and concern arising from the COVID-19 pandemic. For applications of antimicrobial sanitizers to be effective, there is a need to understand limiting factors associated with chemical matrices presented by the relevant applications, ideally supported by understanding of antimicrobial mode-of-action. Here, we present a top-down approach which allows both these critical aspects to be addressed, where chemical understanding then informs mode-of-action characterization. We use the sanitizer electrolyzed water (EW) as an exemplar. Electrolyzed water has been used for water decontamination and surface disinfection of medical equipment for several decades (Hricova et al., 2008). More recent applications have been reported in the food industry, including processing-water sanitization and surface sanitization of factory surfaces and equipment, and of fresh produce (Gil et al., 2015; Kaczmarek et al., 2019). It has been estimated that at least 14% of global food production is lost at the post-harvest level up to (not including) the retail level, with roots, tubers, oil-bearing crops, fruits and vegetables contributing the most to food losses (FAO, 2019). One major contributor to this loss is microbial spoilage, including by molds such as *Aspergillus niger*, a common food spoilage mold found on diverse fruits and vegetables (Taniwaki et al., 2018). A range of preservation methods are used to help mitigate losses to such mold spoilage, but the problem persists. Electrolyzed water can be very effective in killing a range of food-relevant bacteria and fungi (Hricova et al., 2008; Huang et al., 2008). The guidelines for sanitizers to achieve > 5 log reduction (Block, 2001) are usually met with EW for microorganisms in suspension, but lower efficiencies have been reported for certain species or conditions.

Electrolyzed water can be generated using only clean water and salt(NaCl) as substrates, either in two-cell electrochemical systems (to generate acidic and basic EW versions) or in single cell units as with the EW used in this study (Huang et al., 2008; Zhang et al., 2018). Main advantages of EW compared to other sanitizers are that EW allows on-site production from cheap, safe substrates, eliminating the need for transport, handling and storage of concentrated chemical disinfectant, and that it can be inactivated after use simply by mixing with organic matter (Gil et al., 2015). Reducing the usage and chemical contamination of clean water is desirable considering that 29% of the world’s population lack access to safe drinking water and water resources are expected to be negatively impacted by diverse climate change related events (UNESCO, 2019).

Generation of EW involves the formation of free chlorine species (Cl_2_, HOCl, ^–^OCl), and other active compounds at low levels, including ozone (O_3_), chlorine dioxide (ClO_2_), hydrogen peroxide, superoxide and hydroxyl radicals (Jeong et al., 2009; Zhang et al., 2018). Many of the non-chlorine components are unstable. Among the chlorine species found in EW, hypochlorous acid (HOCl) has the strongest antimicrobial effect but dissociates to hypochlorite (^–^OCl) between pH 6.5 and 8.5 and forms Cl_2_ gas at very low pH (<3) (Rahman et al., 2016). The sum of these species is referred to as free available chlorine (FAC, sometimes FC or ACC) (Gil et al., 2015). The FAC in EW can react with inorganic and organic compounds such as ammonia and amino acids (Oomori et al., 2000). Therefore, when applying EW in food production, the organic compounds from food products or in the water or soil could, depending on their concentrations and chemistry, react with the FAC and affect antimicrobial activity. The effect will also depend on the reactivity of such compounds with the different EW components (including non-chlorine species) whose relative contributions to overall EW action are unclear. Compared to conventional sanitation with dissolved hypochlorite salts such as NaOCl, the additional non-chlorine active species can increase the efficacy of EW (Graça et al., 2020). The sanitizer ozonated water is a chlorine-free alternative that is also produced by electrolysis and contains ozone and other reactive oxygen species, but has a lower stability compared to FAC based sanitizers (Baggio et al., 2020; Wang et al., 2020).

Treatment of fungi with FAC or other reactive oxygen species (ROS) promotes oxidative stress and oxidative damage to cellular macromolecules (Avery, 2011; Carmona-Gutierrez et al., 2013). One key molecular target of oxidative stress is iron-sulfur (FeS) cluster proteins (Imlay et al., 2019). FeS-cofactors are found in proteins required for diverse cellular functions, including translation, protein regulation, citric acid cycle, mitochondrial electron transport chain and DNA binding (Cardenas-Rodriguez et al., 2018). Cellular defense from oxidative stress arises through ROS scavenging molecules (antioxidants) and enzymatic systems (Wang et al., 2019). Among the latter, methionine sulfoxide reductases (MSRs) have been shown to help preserve the integrity of FeS-clusters during oxidative stress (Sideri et al., 2009; Alhebshi et al., 2012). The MSR enzymes reduce oxidized methionine (methionine sulfoxide, MetO), effectively functioning as a ROS scavenging system (Brot et al., 1981; Grimaud et al., 2001). The MSRs are highly conserved across evolution (Delaye et al., 2007). In the yeast model of eukaryotes, three different MSRs (MsrA, MsrB, and fRMsr) reduce different MetO isomers including protein-bound MetO (Le et al., 2009). This allows methionine to act as an antioxidant in proteins (protecting other residues from oxidation) and as a regulator of protein activity (Kim et al., 2014).

Here, we investigate the fungicidal activity of EW against the major food spoilage fungus *A. niger*. Results were informed further by mechanistic analyses carried out in the yeast model *Saccharomyces cerevisiae*, also a food spoilage fungus, capitalizing on the extensive understanding and experimental tools available with this organism. The study highlights diverse impacts on EW efficacy of different, incidental organic-material sources. By progressively narrowing down the species of organic material that impact EW activity, we were able to develop and test hypotheses, so revealing new insight to the mechanism of EW action in cells. The outcomes highlight the value of this top-down approach for gaining a comprehensive understanding of how antimicrobial actives may work.

## Materials and Methods

### Fungal Strains and Growth Conditions

The study used the filamentous fungus *Aspergillus niger* N402 (ATCC 64974), and yeast strains *Saccharomyces cerevisiae* BY4741 (*MAT*a; *his3-1*; *leu2-0*; *met15-0*; *ura3-0*), BY4742 (*MAT*α, *his3-1*; *leu2-0*; *ura3-0*; *lys2-0*) and deletion mutants isogenic with the BY4741 parent: *trp1*Δ, *alt1*Δ, *arg4*Δ (from Euroscarf, Germany). The *MSRA* and *MSRB* genes were overexpressed in multicopy plasmids YEp351 and YEp352, respectively, as described previously (Sumner et al., 2005). Overexpression of FeS proteins was with the previously constructed plasmids pCM190-RLI1-HA (Alhebshi et al., 2012) and pCM190-YAH1-HA (Vallières et al., 2017). *Aspergillus niger* was maintained and grown at 28°C on Potato Dextrose Agar (PDA, Oxoid) or YEPD (2% [w/v] bacteriological peptone (Oxoid, Basingstoke, United Kingdom), 1% [w/v] yeast extract (Oxoid), 2% [w/v] D-glucose). Conidia were harvested in 0.1% [v/v] Tween 80 from PDA slopes after 1 week of growth, filtered through a 40 μm cell strainer and spore densities were counted in a hemocytometer. Yeast strains were cultured at 30°C in either YEPD broth or, where indicated, YNB broth (0.69% yeast-nitrogen base without amino acids (Formedium, Norfolk, United Kingdom), 2% [w/v] D-glucose). Amino acids or uracil were added to YNB as needed for strain auxotrophies or plasmid selection. Where necessary, media were solidified with 2% [w/v] agar (Sigma-Aldrich, St. Louis, MO, United States).

### Electrolyzed Water (EW), NaOCl and Ozonated Water

Eloclear^®^ electrolyzed water (EW) was provided by Ozo Innovations (Kidlington, United Kingdom). The EW was manufactured electrochemically in single-cell units with a pH ∼8.7–9.3 and a free available chlorine (FAC) concentration between 1800 and 2000 mg L^–1^, determined (and checked prior to experiments) using the HACH^®^ DPD Free Chlorine Reagent (Permachem^®^) in a HACH^®^ Pocket Colorimeter^TM^ II. EW was stored in plastic containers in the dark at 4°C for up to 2 weeks. Dilutions (v/v) were prepared in HPLC grade water immediately prior to use. NaOCl solution was purchased from ACROS Organics^TM^, Fisher Scientific (5% chlorine). Ozonated water was produced using an Enozo Sanitizing Spray Bottle (SB-100HD) from Enozo Technologies, Inc. (North Andover, MA, United States), kindly provided by GreenTeck Global (Wallingford, United Kingdom). The bottle was filled with sterile filtered tap water (4°C) and ozonated water was produced by spraying for 5 s. The water was used within 30 s (*in vitro* experiments) or 2 min (yeast treatments) of its generation.

### Soil Samples

Soil samples from arable land with varying chemical and textural properties were kindly provided by Hannah Cooper (University of Nottingham). The soil properties are listed in Supplementary Table S1. An additional uncharacterized sample (“soil 8”) was autoclaved soil from an untreated control site at the Woburn long-term sludge experiment, United Kingdom^1^. All samples were sterilized using γ-irradiation for 4 days, corresponding to a total dose of 22–26 kGy. Samples were then dried at 37°C for 4 weeks until a constant weight was reached.

### EW Treatment of *A. niger*

Electrolyzed water was diluted in sterile water to 400–450 mg L^–1^ FAC and either mixed or not with different organics (soil samples, YEPD components, proteins, amino acids). Organics solutions were substituted with sterile water in controls. Five minutes after mixing with EW, the solutions were used to treat conidia (10^5^ spores mL^–1^) at final concentrations of 360–400 mg L^–1^ FAC, ≤0.01% Tween 80. After defined treatment periods (1–7 min), EW treatments were stopped by mixing with an equal volume of two-times concentrated (2X) YEPD. Appropriate dilutions were plated onto YEPD agar and incubated for at least 2 days, 28°C before colony enumeration. For recovery in broth after EW treatment, 100 μL samples were transferred to 96 well plates and incubated statically at 30°C. Growth was determined by OD_600_ readings at appropriate time points in a BioTek^®^ EL800 microplate reader.

### EW Treatment of *S. cerevisiae*

Experimental yeast cultures were inoculated to OD_600_∼0.5 from overnight starter cultures in broth (from single colonies) and cultured to exponential phase (OD_600_∼1.8) in YNB broth. For amino acid pre-culture experiments, different amino acids, S-adenosyl methionine chloride dihydrochloride (SAM, ≥75% purity) or N-acetyl cysteine (NAC) at up to 0.3 mM were included in the experimental cultures. All pre-culture conditions were in buffered YNB (0.1 M potassium phosphate pH 6.0), apart from experiments that included Met pre-culture only. Cells were harvested by centrifugation, washed two times in equal volumes of HPLC grade water, and diluted to OD_600_∼2 in HPLC grade water. Aliquots of 10 μL cell suspension were transferred to 96 well plates, and treated for 5 min with 90 μL of either diluted EW (0.5–1 mg L^–1^ FAC), NaOCl (0.6–0.7 mg L^–1^ FAC) or ozonated water. Treatments were stopped by adding 100 μL of 2X YEPD. Aliquots (100 μL) were transferred to fresh 96 well plates and cultured at 30°C with continuous linear shaking (1096 cycles min^–1^) in 96 well plates in a BioTek microplate reader (Epoch2 or Synergy HTX). The OD_600_ was measured every 30 min and used to estimate survival as described below.

### Estimation of Yeast Survival According to Growth Recovery in Broth

Growth curves from broth cultures after treatments (described above) were analyzed for the period of exponential growth using RStudio. For every six consecutive OD_600_ values during this period (overlapping; i.e., every string of 3 h within the exponential growth period), the exponential regression was determined. The 3-hour period with the highest slope was selected and extrapolation to the y-intercept of the regression was used as an estimate for density of viable cells at *t* = 0 h, similar to the methods described by Fernández-Niño et al. (2018) and Qiu et al. (2017). For validation of the method, *S. cerevisiae* BY4741 was diluted and aliquots spread plated to determine linearity of correlation between values for calculated starting cell density (OD_600_), viability (colony forming units, cfu) and the y-intercept of growth in broth (Supplementary Figures S1A,B). Linearity of the correlation was also examined for cfu (agar) and y-intercept (broth) determinations with EW treated yeast cells. Here, some deviation may be caused by a growth delay of stressed cells (Lu et al., 2009), resulting in lower survival estimates based on the y-intercept compared to cfu enumeration (Supplementary Figures S1C,D). Throughout, EW was diluted to give estimated survival between 5 and 50%. The lower limit was to counter experimental variation observed at very low starting viable-cell densities. The upper limit (50%) was to minimize the relative influence of potential growth-delay effects (as opposed to cell death events) on the measurements: survival rates calculated with the present method are estimates as they may encompass a contribution from any lag phase extension, and will therefore be referred to as recovery rates after treatments.

### Determination of Protein and Humic Acid Contents of Soil Samples

A citrate extraction and a modified Lowry assay were performed as described by Redmile-Gordon et al. (2013). Briefly, 8 mL of 20 mM citrate buffer (pH 7) was added to 1 g of dried soil in 15 mL centrifuge tubes and autoclaved for 30 min (117°C). After autoclaving, the tubes were cooled on ice and then centrifuged (3500 × *g*, 20 min, 4°C). The supernatant was stored at 4°C for the Lowry assay. In an alternative citrate + SDS extraction protocol (Chen et al., 2009), 10 mL of 250 mM citrate buffer (pH 8) was added to 1 g dried soil in 50 mL centrifuge tubes and shaken for 4 h (stuart^®^ see-saw rocker SSL4, 70 rev. min^–1^, with vortexing at 0, 2, and 4 h). After centrifugation (2876 × *g*, 15 min, 4°C), the supernatant was collected as “citrate extract”. 10 mL SDS buffer (1% [w/v] SDS, 0.1 M Tris–HCl (pH 6.8), 20 mM DTT) was added to the pellet, shaken for 30 min (see-saw rocker, as above), and centrifuged to obtain the supernatant (“SDS extract”). Both extracts were combined for the Lowry assay. For the assay, 50 μL extract samples (diluted between 1:5 and 1:12.5 depending on the assay signal of the extract) were transferred to 96 well plates and mixed with 50 μL of PBS containing either BSA as protein standard or a humic acid standard (standard concentrations in PBS: BSA, 40–640 mg mL^–1^; humic acid, 160–640 mg mL^–1^). Assay reagents A and B were prepared as described previously (Redmile-Gordon et al., 2013). To each extract + standard combination, 100 μL of CuSO_4_-containing reagent A (in plate A, for protein and humic acid determination) or of CuSO_4_-free reagent B (in plate B, for humic acid determination) was added. From this point, the standard Lowry protocol (see below) was followed, except that 100 μL 1x Folin-Ciocalteu Phenol reagent (Thermo Fisher) was added. After subtraction of a blank (PBS + reagents), the absorbance corresponding to protein (*Abs*_*prot*_) and humic acid (*Abs*_*hum*_), respectively, were calculated from the absorbance readings for plates A and B (*Abs*_*A*_ and *Abs*_*B*_) (Redmile-Gordon et al., 2013):

Abs=p⁢r⁢o⁢t1.25(Abs-AAbs)BAbs=h⁢u⁢mAbs-B0.2Absp⁢r⁢o⁢t

### Determination of Protein Content in YEPD

The Pierce^TM^ Modified Lowry Protein Assay (Thermo Fisher) was performed according to the manufacturer’s Microplate Procedure. Samples (20 μL of either YEP, peptone or yeast extract, diluted to between 100 and 600 mg L^–1^) were mixed with 20 μL BSA standard (25–750 mg L^–1^). Modified Lowry Reagent (200 μL) was added, before incubation for 10 min in the dark and subsequent addition of 20 μL 1x Folin-Ciocalteu Phenol Reagent. After 30 min incubation in the dark, absorbance at 750 nm was determined in a BioTek^®^ microplate reader (Epoch2).

### *In vitro* Assays With the Fluorescent Dyes APF and HPF

The fluorescent probes hydroxyphenyl fluorescein (HPF) and aminophenyl fluorescein (APF) (Setsukinai et al., 2003) were supplied by Sigma-Aldrich at 5 mM in dimethylformamide (DMF). These were diluted in HPLC grade water to 50 μM. Dye (10 μL of the 50 μM dilution) was mixed with 10 μL water or diluted amino acids (final assay concentration after oxidant addition was 1.2–10 μM), and 80 μL oxidant (diluted EW, NaOCl or pure ozonated water) was added. Fluorescence was recorded immediately (within 2 min of oxidant addition) and at further time points up to 1 h, with excitation at 485/20 nm and emission at 528/20 nm (BioTek^®^ microplate reader Synergy HTX).

### Aconitase and Fumarase Assay in Cell Extracts

The method was as described previously (Lalève et al., 2016). Briefly, *S. cerevisiae* was grown to exponential phase in 50 mL YEPD (250 mL shake flasks). Cells were washed, pooled and treated with EW (except for *in vitro* EW treatments, described below) as outlined above, except that cell suspensions were concentrated to OD_600_∼200 in water and 1 mL suspension was treated with 19 mL EW (5.4–6 mg L^–1^ FAC), with treatments inactivated using 20 mL of 2X YEPD (for survival analysis, samples were taken at this point, diluted and spread to YEPD agar for cfu determination). Cells were harvested (2876 g, 4°C, 10 min), transferred to a 4°C room and resuspended in 250 μL cold resuspension buffer [0.72 mM MnCl_2_ in 10 mM MES (pH 6), Halt^TM^ Protease Inhibitor Cocktail (1x), with oxygen depleted by pre-incubation in 10% CO_2_, 10% H_2_, 80% N_2_ for at least 2 h]. Cells were lysed with 500 μL cold glass beads [acid washed, 425–500 μm (Sigma Aldrich), pre-conditioned with resuspension buffer] and aliquots of the supernatants (50 μL) frozen in liquid nitrogen and stored at −80°C. Protein concentrations in the extracts were determined with the Bio-Rad protein assay kit according to the manufacturer’s Microassay Procedure. For *in vitro* EW treatments, 155 μg protein extracted from untreated cells was mixed with EW (final concentration 180–200 mg L^–1^ FAC) or primaquine (500 μM) for 20 min, in the absence or presence of 3 mM methionine or cysteine. Aliquots comprising 140 μg protein (from *in vivo* or *in vitro* treatments) were then mixed with aconitase buffer (50 mM potassium phosphate buffer [pH 7.4], 30 mM DL-isocitric acid trisodium, 0.6 mM MnCl_2_) or fumarase buffer [50 mM potassium phosphate buffer (pH 7.4), 50 mM L-malic acid] to a total volume of 700 μL in quartz cuvettes. The absorbance at 240 nm was recorded every 30 s for 30 min (baseline correction at 340 nm). Enzyme activities were calculated from the resultant curves as described previously (Lalève et al., 2016).

### Statistical Analysis

Tests for statistical significance were according to a two-tailed paired *t*-test, correcting for multiple comparison by controlling the false discovery rate at FDR < 5%, using a two-stage step-up method (Benjamini et al., 2006). The calculations were performed within GraphPad Prism 8 software. Paired tests were used to control for observed between-experiment variation in EW effect-size. At least three independent replicates were analyzed in each case. Significance was defined by a *p*-value < 0.05. Linear regressions were calculated in Prism 8, to determine *R*^2^ and *p* values.

## Results

### Electrolyzed Water Activity Tolerates High Levels of Added Soil

**FIGURE 1:**
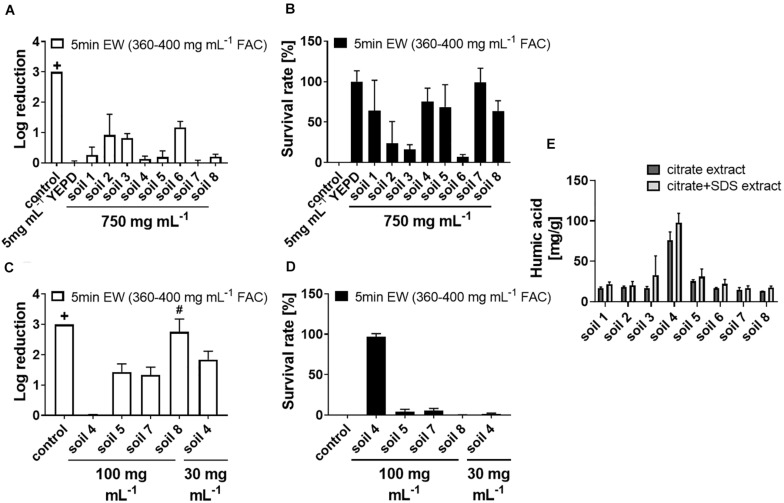
Inactivation of fungicidal EW activity by soil material. Soil (characteristics of soils 1–7 listed in Supplementary Table S1), YEPD or water (control) were added as indicated to EW (400–450 mg L**^–^**^1^ FAC) and incubated for 5 min, before exposing *A. niger* spores to the (soil-supplemented) EW for 5 min, final concentration 360–400 mg L^–1^ FAC. For each experiment, log reduction rates **(A,C)** and survival rates **(B,D)** were calculated from counts of colony growth on YEPD agar after up to 3 days. **(A,B)** Soil added to a final concentration of 750 mg mL**^–^**^1^. **(C,D)** Soil added to final concentrations of 100 or 30 mg mL**^–^**^1^. Data are means ± SD from at least three biological replicates (except soil 8 in A,B: *n* = 2). The log reduction was >3 in control samples (+, no cfu detected when plating ∼2000 spores). In panels **(C,D)** there was >3 log reduction with soil 8 in two out of three biological replicates (#). **(E)** Organic compounds were extracted using methods with either citrate or citrate + SDS and the organic contents in the extracts measured as humic acid using a modified Lowry assay (Redmile-Gordon et al., 2013). Data are means ± SD from two technical replicates.

Sanitizers ideally should have antimicrobial actions resilient to the presence of incidental substances or contaminants. Potential applications of EW in the fresh food industry, among other applications, may be affected by soil residues on produce or other contaminants; a factor that could alter EW activity. Effects of soil on EW activity were therefore tested. In standard experimental conditions, EW at a 20% [v/v] dilution (360–400 mg L^–1^ FAC) was sufficient to kill > 3 log spores of the spoilage mold *A. niger* in 5 min (see “control” in Figure 1A; no colony formation detected when plating ∼2000 spores per plate after EW treatment). Lower EW concentrations allowed greater spore survival (Supplementary Figure S2A). Adding soil to EW treatments (360–400 mg L^–1^ FAC) inactivated EW-dependent killing only at high soil levels (Figure 1A); the soil concentration (750 mg mL^–1^) shown in the figure was chosen after preliminary tests with soil “8” showed that lower soil additions were insufficient for suppressing EW activity. The data are also presented as survival rates in Figure 1B (linear scale) as this helps visualize differences in the sizes of the inactivation effects between the soil types. Four out of eight tested soils did not give full EW inactivation even at 750 mg mL^–1^. The strongest inactivation effect was observed for one soil sample (soil 4) which gave full inactivation of EW at 100 mg mL^–1^ and more limited EW action (<2 log killing) at 30 mg mL^–1^ (Figures 1C,D). The soil concentrations necessary to inactivate EW were compared to a complex organics mix (YEPD, a fungal growth medium). This mix at only 5 mg mL^–1^ was sufficient to inactivate EW fully.

To explain the differences observed for the different soil types, and because YEPD contains high protein levels, the organic content of the soil samples was dissected. The protein content and the humic substance content of the soils were determined in citrate extracts and, to increase the range of extracted proteins, also in citrate + SDS extracts (Chen et al., 2009). Protein levels were very low (below 5–10 mg g^–1^ soil) compared to the humic substance levels and these low protein levels did not correlate with observed EW inactivation efficiency (Supplementary Figure S3). Soil 4 showed a high level of humic substances (in both types of extract) and had the strongest inactivating effect on EW (Figures 1C–E). Humic substances in soil extracts are formed by accumulation, aggregation and degradation of organic molecules during the extraction process (Lehmann and Kleber, 2015). The extraction methods used here do not claim full representation of all soil organics, but the comparison between the soil types indicates a higher organic content in soil 4 compared to the other soil types (see section “Discussion”).

### EW Can Be Inactivated by Proteins and Amino Acids

**FIGURE 2:**
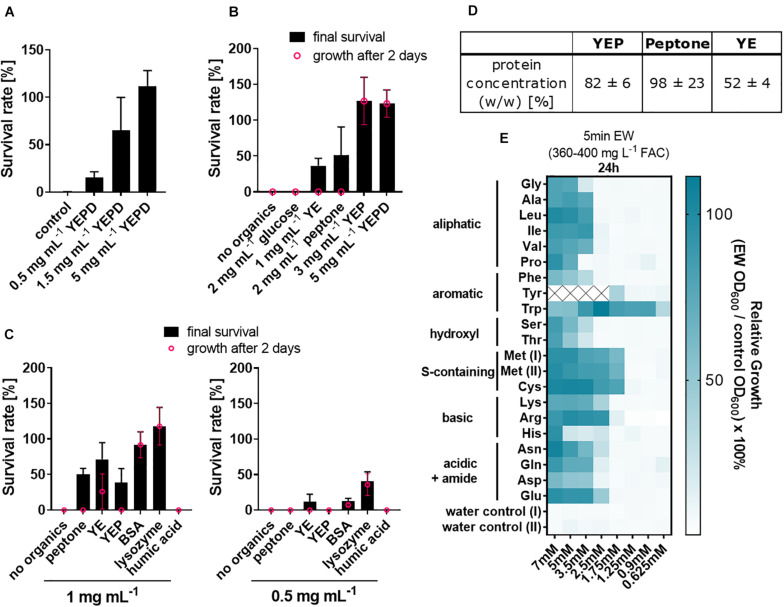
Inactivation of fungicidal EW activity by proteins and amino acids. **(A–C)** Organics were included at the indicated concentrations in EW preparations (360–400 mg L**^–^**^1^ FAC) used to treat *A. niger* spores. **(A)** YEPD [consisting of peptone (40% [w/w] of the dry weight), yeast extract (20%), glucose (40%)], **(B,C)** YEPD components (YE, yeast extract; YEP, YE + peptone; YEPD, YEP + glucose) and **(C)** pure proteins. The organics were added 5 min prior to use of the EW for short-exposure (**A**, 1 min; **B,C**, ∼6 min) treatments of spores. Survival rates were determined by colony counts on YEPD agar. Values shown are means from biological triplicates ± SD. Appearance of colonies was delayed after some treatments, as indicated where % survival after up to a week (black) is greater than from counts at 2 days (pink; growth was at 28°C for the first 2 days and then at RT). **(D)** Protein concentrations in YEP components are means from technical triplicates ± SD. (YEP comprised 66.7% peptone and 33.3% YE, reflecting the relative compositions of these components in YEPD medium). **(E)** Amino acids were included at the indicated concentrations in EW used to treat *A. niger* spores, performed as in A–C. Subsequent growth was determined in YEPD broth by OD_600_ readings after 24 h and normalized to control growth without EW treatment. Mean values are shown from biological triplicates (except lysine, where *n* = 2). Within each replicate experiment, assays were split across two 96-well plates, both of which contained Met as an internal control, shown as Met(I) and Met(II). Tyrosine values ≥2.5 mM are missing due to limited water solubility. Numerical values and standard deviations are listed in Supplementary Table S2.

Because of the above indication of a relationship between soil organic load and EW inactivation, the complex organics mix of YEPD was selected for investigating the effects of different organic constituents of the medium. Increasing additions of YEPD progressively inactivated the fungicidal activity of EW (Figure 2A). YEPD consists of peptone [40% (w/w) of the YEPD dry weight), yeast extract [YE, 20% (w/w)] and glucose [40% (w/w)]. Two of the individual components (peptone and YE) each had inactivating effects on EW, but glucose did not (Figure 2B). Both peptone and YE contain high protein concentrations, determined as 98 and 52% [w/w], respectively, (Figure 2D) (these are estimates as non-protein substances can interfere with the Lowry reaction in complex mixtures). The inactivating effect was then examined with purified proteins (Figure 2C). Lysozyme and bovine serum albumin (BSA) inactivated EW at a concentration range similar to peptone and YE (0.5–1 mg/mL) but achieved a more complete inactivation (∼100% inactivation at 1 mg mL^–1^ BSA or lysozyme). With peptone and YE, colony formation by surviving spores was delayed by up to 4–5 additional days compared to control growth without treatment, whereas with BSA or lysozyme, most colonies were small but visible after the usual 2 days growth period.

To dissect protein constituents that may contribute to EW inactivation, the effects of the protein building blocks, amino acids, were tested. To compare their effects, the amino acid concentrations necessary to inactivate EW were determined at an EW concentration that, on its own, results in > 3 log reduction of *A. niger* spores (360–400 mg L^–1^ FAC). All 20 tested amino acids supplied at between 5 to 7 mM at least partly inactivated fungicidal EW activity (Figure 2E). The strongest inactivating effects on EW (i.e., at amino acid concentrations lower than 2.5 mM) were found for tryptophan (Trp), tyrosine (Tyr, for which 2.5 mM was the highest concentration attainable due to low Tyr solubility in water), cysteine (Cys) and methionine (Met). Certain other amino acids [arginine (Arg), histidine (His), asparagine (Asn)] also showed a mild elevation in EW inactivation compared to the remaining amino acids, evident at later time points (Supplementary Figure S4).

### Growth With High Methionine Levels Increases Resistance of Yeast to Subsequent EW Treatment

**FIGURE 3:**
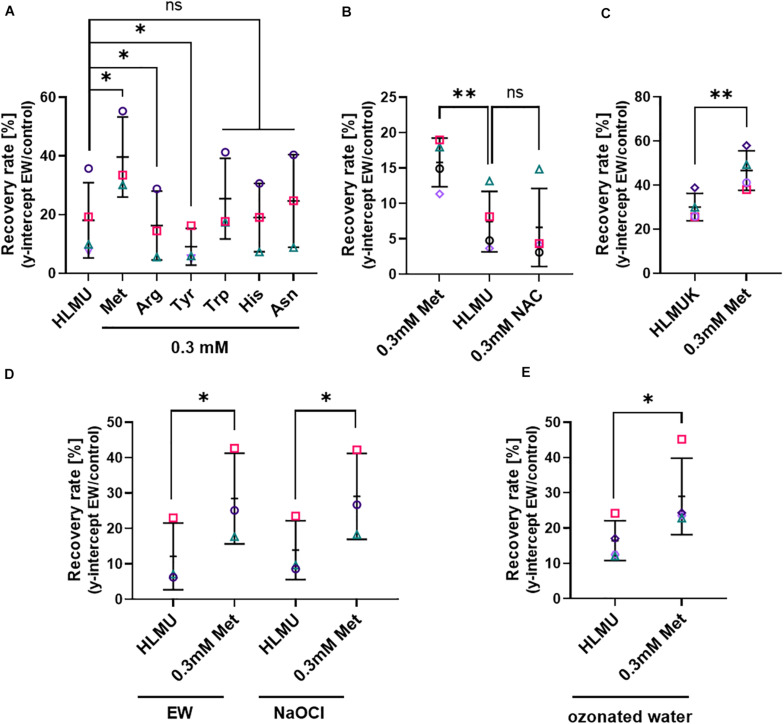
Influence of cellular methionine on recovery of cells after EW, NaOCl and ozonated water treatments. Survival of yeasts after treatment was estimated by subsequent recovery in YEPD broth (see section “Materials and Methods” and Supplementary Figure S1). **(A–C)** Treatment with EW (0.5–1 mg L**^–^**^1^ FAC, 5 min). **(D)** Treatment for 5 min either with EW or NaOCl, in parallel, both oxidants at 0.7 mg L^–1^ FAC. **(E)** Treatment with ozonated water, used within 2 min of generation for a 5 min treatment. Data are shown only for experiments where recovery was >10%, to minimize the influence of technical variation that was observed in response to ozonated water stress. **(A–E)** *S. cerevisiae* BY4741 **(A,B,D,E)** or BY4742 **(C)** were pre-cultured with amino acids at the indicated total concentrations for 4–5 h prior to treatment; control growth and pre-culture were in YNB broth also containing 0.1338 mM Met, 0.129 mM His, 0.763 mM Leu, 0.178 mM Ura (HLMU, **A,B,D,E**), plus 0.21 mM Lys for BY4742 (HLMUK, **C**). Where amino acids were added to the control broth, the pre-culture concentration (0.3 mM) refers to the final total concentration (i.e., including the control-broth content). YNB was buffered (0.1 M potassium phosphate buffer pH 6) in panels **(A,B)**. Cells were washed in water before treatment. NAC, N-acetyl cysteine. All plots: mean values ± SD are shown for at least three biological replicates, with different replicate experiments distinguished by different symbols. Significant differences versus relevant controls are denoted by ^∗^*p* < 0.05, ^∗∗^*p* < 0.01 according to paired *t*-test (two-tailed) with correction for multiple comparisons by controlling the false discovery rate at 5% FDR (Benjamini et al., 2006). ns, not significant.

As certain amino acids were noted above to affect EW activity particularly strongly, it was hypothesized that the cellular content of these amino acids might influence resistance of cells to the killing action of EW activity. This was tested first by supplying higher levels of these amino acids to cells for defined incubation periods followed by removal of any remaining extracellular amino acid before the EW treatment. This was to avoid chemical inactivation of EW by extracellular amino acids, allowing study of the effect of amino acid accumulated by cells. These experiments were carried out in *Saccharomyces cerevisiae* because amino acid uptake among the fungi is best characterized in this yeast model, for which appropriate auxotroph strains and other resources are available, allowing more extensive *in vivo* studies of EW mode of action. Lower EW concentrations (0.5–1 mg L^–1^ FAC) than in the previous experiments were needed with *S. cerevisiae* to avoid complete killing, so that the fungal response to EW could be studied (see Supplementary Figure S2B for dose-dependent yeast survival after EW treatment). Survival of *S. cerevisiae* was estimated as recovery by culturing cells in broth after EW treatment and determining the y-intercept of the exponential phase as an estimate for the starting viable-cell density (see section “Materials and Methods” and Supplementary Figure S1). Exogenously supplied amino acids can be readily taken up by yeast cells. Pre-culture of yeast for 4–5 h in defined medium (YNB) supplemented with different amino acids (0.3 mM), followed by EW treatment in the absence of amino acids, revealed a protective effect of methionine (Figure 3A). Pre-culture with Met increased recovery after EW treatment by >1.5-fold (FDR adjusted *p* = 0.0242). Other amino acids chosen based on the EW inactivation results above (Trp, Tyr, Arg, His, and Asn) did not exhibit a protective effect (Arg and Tyr actually gave slight sensitization). Pre-culture with Cys supplement inhibited yeast growth so it could not be tested (data not shown). Pre-culture with N-acetyl-cysteine (NAC), commonly used as a Cys precursor, did not inhibit yeast growth but did not protect against EW (Figure 3B). The protective effect of Met was not dependent on the Met auxotrophy (biosynthetic defect demanding externally supplied Met) of the *S. cerevisiae* BY4741 laboratory strain used above, as the isogenic Met-prototrophic strain *S. cerevisiae* BY4742 was also protected by Met pre-treatment (Figure 3C). Furthermore, deletion strains auxotrophic for Trp (*trp1*Δ), Arg (*arg4*Δ) or His (BY4741: *met15*Δ, *his3*Δ), and the partial alanine auxotroph *alt1*Δ (Ala did not have a particularly strong EW inactivation effect [see previous section] and was used as a control amino acid), were not protected by their respective amino acid (Figure 3A and Supplementary Figure S5).

Because EW is likely to contain different active species, we tested whether the observed effect of Met pre-culture on resistance to EW (Figures 3A–C) was mimicked with NaOCl treatment. Similar to EW, pre-culture with Met gave increased resistance to NaOCl treatment (Figure 3D). Like EW, ozonated water was produced using an electrolysis technology but, unlike EW, it does not contain FAC. In water, ozone reacts to form other reactive species such as highly reactive hydroxyl radicals (Wang et al., 2020). Recovery after ozonated water treatment was also increased after Met pre-culture (Figure 3E). The results indicate that Met may protect against both chlorine and non-chlorine active species in EW.

### The Protective Effect of Met Pre-culture Is Probably Not Due to a Downstream Product of Met Metabolism

**FIGURE 4:**
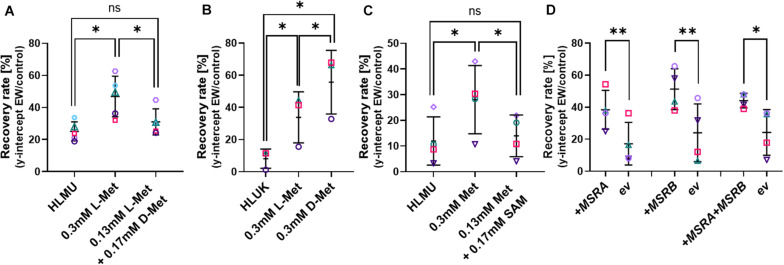
Influence of methionine isomers, SAM and MetO-reduction on recovery of cells after EW treatment. Survival of yeasts after EW treatment (0.5–1 mg L**^–^**^1^ FAC, 5 min) was estimated by subsequent recovery in YEPD broth (see section “Materials and Methods” and Supplementary Figure S1). **(A–C)** Pre-culture of *S. cerevisiae* BY4741 **(A,C)** or BY4742 **(B)** with L-Met, D-Met or S-adenosyl-methionine (SAM) at the indicated total concentrations for 4–5 h prior to EW treatment; control growth and pre-culture were in YNB broth also containing 0.1338 mM Met, 0.129 mM His, 0.763 mM Leu, 0.178 mM Ura (HLMU, **A,C**), or 0.21 mM Lys instead of Met for BY4742 (HLUK, **B**). Where amino acids were added to the control broth, the pre-culture concentration (0.3 mM) refers to the final total concentration (i.e., including the control-broth content). YNB was buffered (0.1 M potassium phosphate buffer pH 6) in C. Cells were washed in water before EW treatment. **(D)** Overexpression of *MSRA* and *MSRB* in multicopy vectors YEp351 and YEp352, respectively (ev, empty vector), in *S. cerevisiae* BY4741. All plots: mean values ± SD are shown for at least three biological replicates, with different replicate experiments distinguished by different symbols. Comparisons of interest were tested by paired *t*-test (two-tailed) with correction for multiple comparisons by controlling the false discovery rate at 5% FDR (Benjamini et al., 2006). ^∗^*p* < 0.05; ^∗∗^*p* < 0.01; ns, not significant.

All cellular life relies on L-isomers of amino acids, and we confirmed that *S. cerevisiae* BY4741 is unable to grow on D-Met as the sole Met source, while growth in medium containing L-Met was not affected by D-Met addition (Supplementary Figure S6). Adding D-Met to medium containing L-Met (0.13 mM) to a total of 0.3 mM Met (D + L) did not mimic the protective effect observed with 0.3 mM L-Met in the Met-auxotrophic strain BY4741 (Figure 4A). In contrast, adding D-Met to medium free of L-Met for the pre-culture of the Met prototroph strain BY4742 resulted in a protective effect; the effect of D-Met was actually larger than the effect of L-Met pre-culture (Figure 4B). There could be increased D-Met uptake in medium free of L-Met due to a lack of competition for Met uptake systems (such as Mup1) in the absence of L-Met (Gits and Grenson, 1967). This protective effect of the metabolically inactive D-Met suggests a direct role of Met itself in EW resistance.

Saturation of Met uptake is reported to occur within 10 min of Met addition to Met-free medium (Schwabe and Bruggeman, 2014), and downregulation of high-affinity Met uptake systems in response to high Met also commences within 10 min (Menant et al., 2006). Here, 10 min L-Met pre-culture was sufficient to increase resistance of yeast to EW, and longer Met pre-treatments did not add further advantage (Supplementary Figure S7). The first step of downstream methionine metabolism yields S-adenosyl-methionine (SAM). Pre-treatment with SAM did not improve resistance to EW, further supporting a role of Met itself in protection, rather than a Met metabolic product (Figure 4C). SAM uptake and utilization was confirmed by the restoration of growth of *S. cerevisiae* BY4741 in Met-free, SAM-supplemented medium (Supplementary Figure S6).

### The Protective Effect of Methionine May Arise From Oxidation of Reduced Met by the FAC in EW

Methionine residues can be naturally oxidized to methionine sulfoxide (MetO) but can be reduced back to methionine by cellular methionine sulfoxide reductases, encoded by *MSRA*, *MSRB*, and *fRMSR* in yeast. It was hypothesized that Met oxidation by EW could explain the above protective effects on cells of adding Met in its reduced form, suggesting a potential role of accumulated Met as a direct ROS scavenger in EW stress. Accordingly, improved maintenance of reduced Met by MSR activity should also increase resistance to EW. The yeast MSRA and MSRB enzymes were overexpressed on multicopy plasmids as characterized previously (Sumner et al., 2005), either alone or in combination. In all cases, resistance of yeast to EW was significantly increased by elevated MSR expression (Figure 4D), indicating that the level of reduced Met specifically is important for resistance to EW.

**FIGURE 5:**
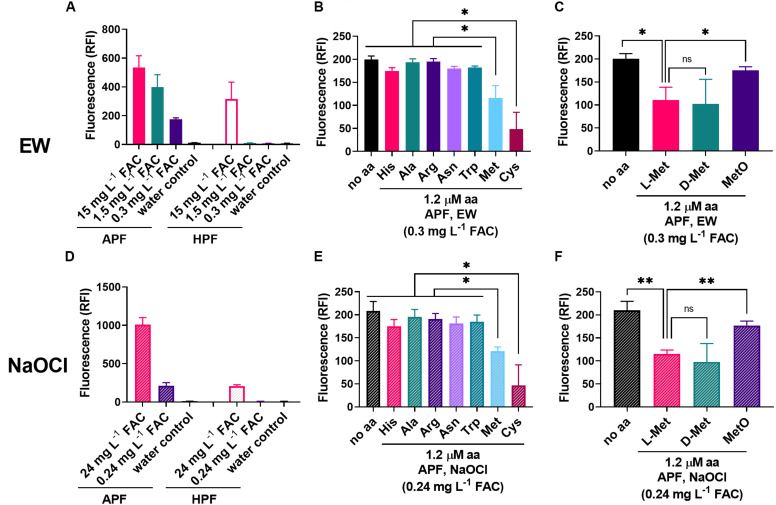
Influence of amino acids on the oxidizing properties of EW and NaOCl. **(A,D)** The fluorescent ROS probes APF and HPF (5 μM) were mixed with different dilutions of EW **(A)** or NaOCl **(D)** [100% (v/v) EW = 1.8–2.0 g L**^–^**^1^ FAC; 100% NaOCl = 3.0–3.1 g L^–1^ FAC]. Fluorescence (RFI, relative fluorescence intensity) was recorded within 2 min of mixing. **B,C,E,F**) The effect of amino acids (1.2 μM) present in the APF-oxidant mix on the fluorescence signal was tested at approximately equimolar concentrations of dye (5 μM) and EW [∼0.3 mg L**^–^**^1^ FAC ∼5.5–6.1 μM HOCl (FAC expected to be primarily HOCl); **B,C**], or NaOCl [∼0.24 mg L**^–^**^1^ = 3.2–3.3 μM; **E,F**]. Mean values ± SD are shown for at least three biological replicates. ^∗^*p* < 0.05, ^∗∗^*p* < 0.01, ns, not significant; according to paired *t*-test (two-tailed) with correction for multiple comparisons by controlling the false discovery rate at 5% FDR (Benjamini et al., 2006).

Methionine might be directly oxidized by EW, with simultaneous depletion of oxidizing species in EW. The oxidizing properties of EW were assayed with the oxidation-sensitive fluoroprobes HPF and APF. These probes enable the distinction between FAC (^–^OCl/HOCl) and other ROS (mainly ^•^OH and ONOO^–^). While the reactivity of APF and HPF with other ROS is of a similar order (albeit up to 5x stronger with APF), APF reactivity with FAC is up to 600× stronger than HPF (Setsukinai et al., 2003). HPF fluoresces strongly only at ≥5-fold excess of FAC (Flemmig et al., 2012). To test selective detection of FAC with APF but not HPF, equimolar levels of probe and oxidant were used (5 μM probe; the FAC of EW would correspond to ∼5.5 μM HOCl at ∼0.3 mg L^–1^ FAC). Only APF was fluorescent at these levels, whereas EW concentrations ≥∼1.5 mg L^–1^ FAC resulted in both APF and HPF fluorescence (Figure 5A). A similar pattern was observed for diluted NaOCl (Figure 5D), indicating that the FAC might be (one of) the main oxidizing agent(s) in the EW. Other ROS probes were tested but resulted either in no signal (DHE) or no signal at equimolar levels of probe and oxidant (H_2_DCFDA; when mixed with undiluted EW, an unstable fluorescence signal was obtained that decreased within minutes, indicating potential oxidative damage to the probe at high FAC; not shown).

The amino acids His, Ala, Arg, Asn, Trp, Met, and Cys (chosen based on a stronger EW inactivation effect compared to other amino acids, see Figure 2E, plus Ala as a negative control) were added to the EW and NaOCl oxidant solutions, to test effects on APF oxidation. Only methionine and cysteine (at 1.2 μM) produced significant decreases in the APF-oxidizing properties of either EW or NaOCl (Figures 5B,E). Because the work described above indicated that L-Met (reduced) was more important for increased resistance of yeast to EW than MetO (oxidized Met, substrate of MSR enzymes) but not D-Met, these molecules were compared also for effects on APF oxidation by EW and NaOCl (Figures 5C,F). L-Met and D-Met resulted in an equally strong suppression of the oxidizing properties of both EW and NaOCl, consistent with the mediation of a protective effect in yeast by either isomer (Figure 4B). MetO had a weaker effect on APF oxidation by EW and NaOCl, in keeping with a model in which oxidation of Met to MetO by EW or NaOCl quenches additional oxidation of APF.

**FIGURE 6:**
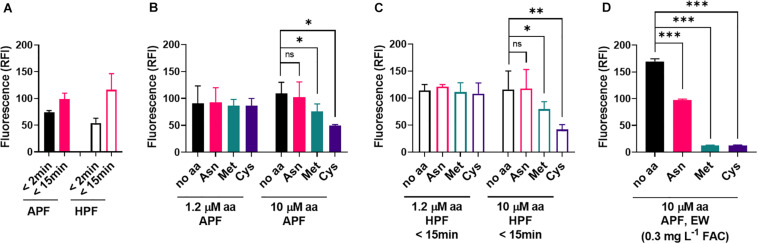
Influence of amino acids on the oxidizing properties of ozonated water. **(A)** The fluorescent ROS probes APF and HPF (5 μM) were mixed with ozonated water. Fluorescence (RFI, relative fluorescence intensity) was recorded within 2 min of mixing and again 13 min later. **(B,C)** Effects of amino acids supplied at the specified concentrations in the APF-ozonated water mix **(B)** or HPF-ozonated water mix **(C)**. Because HPF fluorescence strongly increased within the first few minutes, and no significant effect of the amino acids was visible at the first time point (<2 min), data for the 15 min time point are shown for HPF. **(D)** Effect of high amino acid concentrations (10 μM) supplied in an APF-EW mix (experimental details as described in Figure 5). Mean values ± SD are shown for at least three biological replicates. ^∗^*p* < 0.05, ^∗∗^*p* < 0.01, ^∗∗∗^*p* < 0.001, ns, not significant; according to paired *t*-test (two-tailed) with correction for multiple comparisons by controlling the false discovery rate at 5% FDR (Benjamini et al., 2006).

In contrast to EW, ozonated water produced similar fluorescence responses with APF and HPF (Figure 6A). Met and Cys could suppress these oxidizing actions of ozonated water, but this required higher concentrations of the amino acids (10 μM) than for EW inactivation (1.2 μM) (Figures 6B,C). At 10 μM amino acid, fluoroprobe oxidation by EW was fully inactivated by Met and Cys and partially inactivated by Asn (Figure 6D). These results suggest a lower reactivity of the amino acids with ozonated water compared with EW (Figures 5, 6). The strong reactivity of EW with Met and Cys suggests that the FAC, as opposed to other potential ROS components of EW, is likely to be the main EW component reacting with these amino acids.

### EW Treatment Affects FeS Cluster Proteins

**FIGURE 7:**
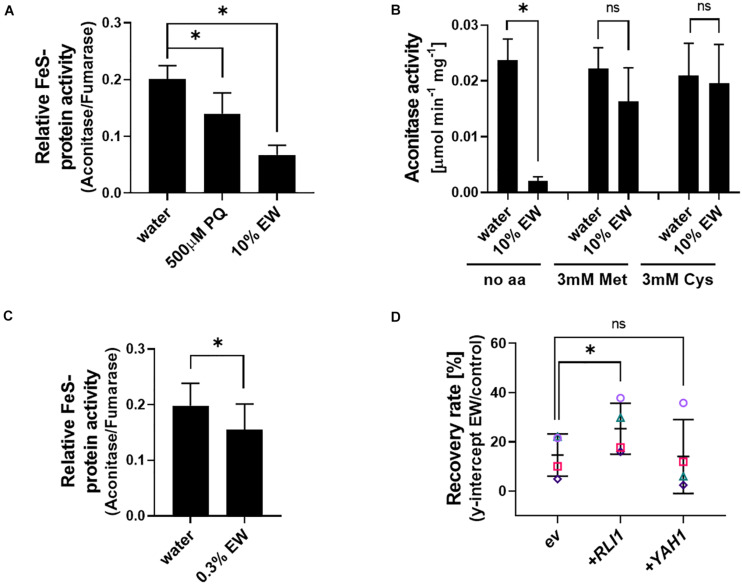
Relationship between EW action and FeS protein activity. **(A)** Crude protein extracts from exponentially growing *S. cerevisiae* BY4741 cells were treated *in vitro* with 500 μM primaquine (PQ), 10% EW [v/v] (180–200 mg L**^–^**^1^ FAC) or water (control) for 20 min, before determination of aconitase and fumarase activities. Aconitase activity is normalized to fumarase activity (a non-FeS protein); for absolute activities, see Supplementary Figure S8. **(B)** Aconitase activity of *in vitro* treatments performed as in panel **(A)** but with inclusion of 3 mM Met or Cys added to protein extracts shortly before EW exposure. **(C)** *In vivo* EW treatment (0.3% EW: 5.4–6 mg L^–1^ FAC) of exponentially growing yeast cells followed by protein extraction and determination of enzyme activities (the dense yeast suspensions needed for sufficient protein yield required a higher % EW here for comparable effect to other *in vivo* treatments in this study). **(D)** Overexpression of the essential FeS proteins *RLI1* or *YAH1* from vector pCM190 (ev, empty vector) in *S. cerevisiae* BY4741. Survival of yeasts after EW treatment (0.5–1 mg L^–1^ FAC, 5 min) was estimated by subsequent recovery in YEPD broth (see section “Materials and Methods” and Supplementary Figure S1). Mean values ± SD are shown for at least three biological replicates, with different replicate experiments distinguished by different symbols in panel **(D)**. ^∗^*p* < 0.05, ns, not significant; according to paired *t*-test (two-tailed) with correction for multiple comparisons by controlling the false discovery rate at 5% FDR (Benjamini et al., 2006).

Methionine in its oxidized state has previously been reported to increase oxidative damage to iron-sulfur clusters (Sideri et al., 2009). We hypothesized that the protection against EW by reduced-Met observed here could be related to the maintenance of FeS protein function. Protein extracts from yeast were exposed to EW, with primaquine treatment serving as a positive control as this drug is known to target FeS clusters (Lalève et al., 2016). Relative activity of the FeS cluster protein aconitase (normalized to fumarase activity) was decreased by ∼70% in EW treated extracts (Figure 7A; absolute activities are shown in Supplementary Figure S8; fumarase is also a citric acid cycle enzyme but does not contain an FeS cluster). As fumarase activity was not significantly affected by EW (Supplementary Figure S8), aconitase only was assayed in further *in vitro* experiments, at standardized protein-extract additions. Adding Met or Cys to the *in vitro* EW treatments suppressed the strong inhibition of aconitase activity by EW (Figure 7B).

To test whether similar effects could be detected during *in vivo* EW treatment of yeast cells, an EW dilution yielding >80% cell viability (according to cfu counts) was used to avoid non-specific effects due to lethality. Enzyme assays with protein extracts obtained from the EW-treated cells revealed a ∼22% decrease in relative aconitase activity (*p* = 0.0425) (Figure 7C). A different FeS protein, the essential Rli1 protein, has roles in critical cellular functions such as translation, ribosome biogenesis and recycling (Kispal et al., 2005; Schuller and Green, 2017). It is an important target of oxidative stress due to impaired supply of the FeS-cofactor to the protein under oxidative stress conditions, and Rli1 overexpression in yeast increases resistance to pro-oxidants (Alhebshi et al., 2012). Overexpression of Rli1 increased yeast resistance to EW (Figure 7D). In contrast, overexpression of a different essential FeS-protein, Yah1, did not reproducibly increase resistance to EW, consistent with the reported phenotype of this particular overexpression strain under pro-oxidant stress (Vallières et al., 2017). The results suggest that FeS cluster proteins are an important cellular target of EW action.

## Discussion

Sanitizers are vital products for managing microbial contamination in diverse applications, including in food processing, medical facilities, water treatment and, increasingly, personal use (Huang et al., 2008; Gil et al., 2015). Key considerations determining sanitizer efficacy in particular uses include: (a) the potential for incidental dampening of activity, e.g., by contaminating organic matter, and (b) the mode of antimicrobial action, so potential resistance among target microorganisms can be understood and anticipated. The top-down approach that we introduced here, with the sanitizer EW, enabled us to shed new light on both these key aspects; so providing comprehensive new understanding of EW action and efficacy. Our findings that incidental protein and particular amino acids strongly affect the fungicidal EW activity then led us to new understanding of the oxidizing mode of EW action, in which reduced-methionine of cells plays a critical role.

In the case of processing applications relevant to the food industry, sanitation may be influenced by soil and complex organic mixtures derived from fresh produce or processing and irrigation water. It has previously been reported that different complex organics (peptone, glycine, milk, minced meat, chopped cabbage, and river natural organic matter) can react with the FAC in EW, depleting the FAC and forming combined chlorine with lower sanitation activity compared to FAC (Oomori et al., 2000; Ogunniyi et al., 2019). Combined chlorine compounds such as chloramines (from reaction of FAC with amines) can retain mild oxidizing activity (Hawkins et al., 2003). This highlights the importance of survival-based tests compared to FAC analysis for a more complete assessment of EW efficacy. In this study, survival was assessed by recovery in complex media, meaning that sub-lethal damage to cells and spores cannot be ruled out, previously reported for EW treatments (Lan et al., 2019). This may partly explain the growth delay observed after EW treatment in some conditions (Figure 2).

Unexpectedly large additions of soil were needed to decrease the fungicidal activity of EW in this study, although this resonates with the high levels of NaOCl (6%) used to remove organic compounds from soil samples (Margenot et al., 2015). This indicates that EW can be used for sanitization in the presence of moderate levels of soil, or even for the sanitization of soil samples (Harvey et al., 2020). Reactive oxygen species can be scavenged in soil by plant-derived tannins and other phenolic compounds (Rimmer, 2006). The FAC, hydrogen peroxide or other ROS present in EW could be inactivated after direct oxidation of soil organics, or reaction with inorganic metal species [hydroxyl radical formation involving H_2_O_2_ (Fenton chemistry) or HOCl], could lead to further reactivities with aromatic and other organic compounds (Mikutta et al., 2005; Panasenko et al., 2013). Accordingly, the soil type with the highest organics content in this study (a sandy clay loam soil) had the strongest inactivating effect on EW. Its high organics content was supported both by analyses performed here, as well as a higher loss-on-ignition value (LOI, an indicator of soil organic matter) (Supplementary Table S1). Previously, the bactericidal activities of EW and NaOCl were reported to be inactivated to a similar extent by complex natural organics (Ogunniyi et al., 2019). While certain soil organics are targets of NaOCl (sterols, long-chained lipids, lignin dimers), other soil compounds are less reactive (Sleutel et al., 2009). The latter finding was explained by potential protective effects by the soil minerals and structure that may shield molecules such as peptides from NaOCl exposure. Interestingly, a moderate (but not significant) correlation between decreasing particle size and EW activity (spore inactivation) was apparent for the soil samples tested here [Pearson’s *r* values for spore survival versus silt (smaller) or sand (larger) particles were 0.48 or −0.52, respectively]. This is consistent with less shielding of cells or molecules in smaller particles. It is clear from the results that some fungicidal activity of EW persisted at moderate levels of soil contamination, although this could be coincident with the formation of by-products of concern, such as trihalomethanes (THMs) from the reaction of soil organics with the FAC (Jackman and Hughes, 2010).

Previously, partial or full inactivation of EW occurred over a wide concentration range for different organics (0.04–100 g L^–1^) (Oomori et al., 2000; Jo et al., 2018; Ogunniyi et al., 2019), underscoring the need to understand further EW reactivity with different chemical components of complex organic mixes. Inactivation of the FAC and the bactericidal activity of EW (31–50 mg L^–1^ FAC) has been reported in 0.1–1 g L^–1^ peptone (Oomori et al., 2000; Jo et al., 2018), and we found comparable reactivity of the present EW (360–400 mg L^–1^ FAC) with ∼1 g L^–1^ peptone, yeast extract or pure proteins, according to fungicidal activity. As proteins are highly abundant in all living organisms and in many foods or as surface-contaminants, these are important considerations for EW applications.

The reactivity of protein components with FAC (HOCl/^–^OCl, at pH 7.4) is reported to decrease in the order: Cys > Met > cystine ≈ His ≈α-amino group (in free amino acids) ≈ terminal amino group (in proteins) > Trp > Lys > Tyr ≈ Arg > backbone amides > Asn ≈ Gln (Pattison and Davies, 2001; Storkey et al., 2014). Here, amino acids with the strongest EW-inactivation effects were Met, Cys, His, Trp, Tyr, Arg, and Asn, with Met and Cys found to suppress most strongly the oxidizing properties of EW and NaOCl. The results were in close agreement with the reactivity data for FAC, suggesting that FAC in EW could at least partly account for EW reactivity with (and inactivation by) protein and amino acids. Non-chlorine products of electrolysis were tested here by using ozonated water. Amino acid reactivity of ozone is reported as Cys > Trp ≈ Met > Phe ≈ His (Sharma and Graham, 2010). The rate constants for Cys and Met reactivity with ozone are of the order 10^6^ M^–1^ s^–1^ (pH 8) whereas reactivities of Met and Cys with FAC are of the order 10^7^−10^8^ M^–1^ s^–1^ (pH 7.4) (Storkey et al., 2014). This supports the present data, indicating that Met and Cys have greater effects with NaOCl and EW than with ozonated water, consistent with a primary role for the FAC in the reactivity of EW with these amino acids.

Several amino acids are known to be especially prone to oxidation, such as Pro, Arg, Lys, Thr, Trp, Phe, Tyr, Cys, and Met (Baraibar et al., 2013). However, the data presented here on EW reactivity and amino acids matches better the specific reactivities of FAC (we observed little particular effect with Pro, Lys, Thr or Phe). While non-chlorine ROS may contribute to the antimicrobial effect of EW, a major role is less likely in stored EW solutions (as used here) due to the short life time of these ROS (Jeong et al., 2007). Within cells, FAC species may also lead to formation and conversion of other ROS species which arise as respiratory by-products: Hypochlorous acid reacts with superoxide radicals and with iron to form hydroxyl radicals, and with hydrogen peroxide to form singlet oxygen (Panasenko et al., 2013). Hydroxyl radicals are highly reactive and can oxidize most cellular molecules, including all proteinogenic amino acids (rate constants > 10^7^ M^–1^ s^–1^), the most reactive of which include Met and Cys (>10^9^ M^–1^ s^–1^) (Xu and Chance, 2005).

The reaction of FAC with proteins may lead to protein damage (aggregation, fragmentation, misfolding, cross-linking) and side-chain damage (e.g., formation of chloramines, carbonyls), but also to enhanced activity of hypochlorite-responsive chaperone proteins (Pattison and Davies, 2001; Cater et al., 2019). Protein damage after FAC treatment has been reported in diverse cell types (Winter et al., 2008; Carmona-Gutierrez et al., 2013), and metabolome studies revealed that EW affects amino acid levels and associated pathways in bacteria (Liu et al., 2017, 2020), consistent with the high reactivity of EW with amino acids discussed above. The data presented here indicate a potential protective system against EW-induced damage *in vivo*, involving methionine. Pre-culture with methionine, to allow accumulation of the amino acid, specifically (compared to other amino acids) increased recovery from EW treatment. The effect could be mimicked by the D-Met isomer but not by the first metabolic product of Met utilization (SAM), and occurred rapidly (within 10 min incubation with elevated Met). This suggested a role of the methionine molecule itself in protection against EW. Furthermore, the Met protective effect appeared to extend to a more general form of oxidative stress protection, as it could be reproduced with both NaOCl and ozonated water. As the production of EW yields both FAC and non-chlorine reactive oxygen species (Jeong et al., 2009; Zhang et al., 2018), the results suggest that Met may protect against both types of active species. One possible explanation is that free or protein-incorporated Met acts as a ROS scavenger during EW treatment. Methionine residues in proteins are considered to confer a ROS scavenging action that may protect other residues from oxidative damage (Schindeldecker and Moosmann, 2015). In addition, reversible oxidation of Met residues regulates the activity of different proteins (Drazic et al., 2013; Nicklow and Sevier, 2020), while Met mis-incorporation into proteins may offer short-term protection against oxidative stress (Netzer et al., 2009; Lee et al., 2014). The proposed role of Met in ROS scavenging is supported by the Met/MetO recycling system, where methionine sulfoxide reductases (MSRs) re-reduce oxidized methionine (Brot et al., 1981; Grimaud et al., 2001). Here, MSR overexpressing yeast cells had increased resistance to EW. We also observed several parallels between EW and NaOCl and, elsewhere, HOCl produced by neutrophils as part of the immune response increased Met oxidation in *E. coli* while elevated MSR expression gave increased bacterial survival of HOCl (Rosen et al., 2009). Replacing Met residues with norleucine in *E. coli* proteins increased the bacterium’s sensitivity to NaOCl, supporting the hypothesis that Met residues might protect proteins from FAC stress (Luo and Levine, 2009). In yeast, deletion of MSRA or double deletion of MSRA/MSRB increased NaOCl sensitivity, and overexpression of MSRA or Met-rich proteins protected against NaOCl (Tarrago et al., 2012), similar to the protective effect of MSR overexpression reported here. Interestingly, other amino acids (Trp, Tyr) that were as or more reactive with EW compared to Met did not provide protection when supplied to yeast prior to EW treatments. The lack of a specific oxidation repair system for these amino acids (such as the MSR system for Met) may explain why accumulation of non-Met amino acids does not improve yeast resistance to EW.

Increased oxidant resistance has recently been described after pre-culture of yeast cells with high lysine, similar to the methionine pre-culture here (Olin-Sandoval et al., 2019). However, lysine pre-culture did not improve EW recovery in this study (data not shown). The previously reported lysine effect was explained by potential regulatory effects of excess Lys entering polyamine metabolism, and by increased production of the ROS scavenger glutathione. The protective effect of Met pre-culture against EW may also, at least partly, be more indirect than purely ROS scavenging. It has been shown previously that elevated MetO levels or decreased reduced-Met lead to increased oxidative targeting and turnover of FeS clusters (Sideri et al., 2009). Accordingly, increasing the level (via pre-culture) or maintenance (MSR overexpression) of reduced Met should help to keep FeS clusters intact in oxidizing cellular environments. Oxidative stress in general is well known to target surface-exposed FeS clusters in proteins (Imlay et al., 2019). FeS proteins are highly reactive with FAC (Albrich et al., 1981) and NaOCl may, potentially via ROS generation in cells, damage the clusters in bacterial FeS proteins (Romsang et al., 2018). This study shows that EW impairs activity of the FeS cluster protein aconitase, a known target of pro-oxidant agents (Lalève et al., 2016). Rli1 overexpression protected against EW, similar to a reported protective effect against several pro-oxidants (Alhebshi et al., 2012). Rli1 is required for the nuclear export of ribosomal subunits, initiation and termination of translation and ribosome recycling (Kispal et al., 2005; Schuller and Green, 2017). Rli1 function is highly conserved and essential for cells, requiring the mitochondrial and cytosolic FeS assembly machinery to provide its two [4Fe-4S] clusters, pathways that are ROS hyper-sensitive (Gomez et al., 2014; Paul et al., 2015). This study suggests that Rli1 could be a key target of EW, advancing our understanding of EW mode of action. The reported contribution of reduced Met to FeS cluster maintenance (Sideri et al., 2009) offers a further explanation for the protection by Met against EW, in addition to the potential ROS-scavenging mechanism discussed above.

## Conclusion

Using a top-down approach leading from complex matrices to cellular targets, we show the impact of protein levels and amino acids (especially methionine and cysteine) on fungicidal efficacy of EW, which most likely arises from inactivation of the FAC in EW by these molecules. We also show that methionine plays a key role in the mechanism of EW action in fungal cells, as a direct ROS scavenger and/or as a protector of other primary ROS targets. Increasing the level of reduced Met, either by enhancing MetO reduction or by feeding more Met, may protect FeS cluster proteins. We show that FeS cluster proteins, essential for cell viability, may be major targets of EW. This work provides important insight into the mode of action of chlorine based sanitizers, and potentially other pro-oxidant products. Such knowledge could inform rational combination of EW with other treatments (often referred to as “hurdle” technology in the food industry) targeting complementary functions or processes in organisms. Furthermore, the data may help to understand and predict antimicrobial efficacy against different spoilage or pathogenic microorganisms in diverse industry or domestic settings. The data are particularly relevant in regions of the world where EW use relies on water sources that contain high levels of constituents such as the compounds identified here that inactivate EW. The results establish the benefits of a comprehensive top-down approach for understanding sanitizer activity, leading from the industrial application right down to molecular targets.

## Data Availability Statement

All datasets presented in this study are included in the article/Supplementary Material.

## Author Contributions

FW: experimentation and data analysis and manuscript original draft. FR, IS, RG, and SA: conceptualization, funding acquisition and supervision. RG and SA: project administration. FR, FW, IS, RG, and SA: manuscript review and editing. All authors contributed to the article and approved the submitted version.

## Conflict of Interest

The authors declare that the research was conducted in the absence of any commercial or financial relationships that could be construed as a potential conflict of interest.

## Footnotes

1 http://www.era.rothamsted.ac.uk/Other#SEC11
