# Supplementary material for: Top-Down Characterization of an Antimicrobial Sanitizer, Leading From Quenchers of Efficacy to Mode of Action
Source: Front Microbiol. 2020 Sep 25;11:575157. doi: 10.3389/fmicb.2020.575157 (PMC7546784; doi:10.3389/fmicb.2020.575157)
Supplement: Supplementary file 1 [file Presentation_1.pdf]

SUPPLEMENTARY DATA

Wohlgemuth et al

Table S1: Soil type characterisation.

#	Particle Size Characterisation	LOI[a] [%]	Sand [%]	Silt [%]	Clay [%]	pH
1	Sandy_Silt_Loam	7.76	24.6	64.4	10.9	7.71
2	Sandy_Clay_Loam	7.66	66.8	12.6	20.6	7.9
3	Sandy_Clay_Loam	3.82	65.8	10.6	23.6	7.88
4	Sandy_Clay_Loam	13.68	59.3	21.7	18.9	7.73
5	Clay	7.78	34.4	30.5	35.1	6.38
6	Sandy_Clay_Loam	6.18	66.8	12.6	20.6	7.71
7	Sandy_Clay_Loam	5.85	55.5	24.3	20.2	8.1

[a]LOI (loss on ignition) is a measure for organics content.

Table S2: Related to Figure 2E. Inactivation of fungicidal EW activity by amino acids.

Amino acid	Gly		Ala		Leu		Ile	
conc.	mean[a]	SD	mean	SD	mean	SD	mean	SD
7mM	79.73	3.38	80.23	7.15	100.53	13.08	87.92	7.18
5mM	76.70	11.32	85.25	11.19	96.81	11.91	86.45	7.33
3.5mM	21.39	17.69	75.88	12.99	84.63	14.86	90.34	6.18
2.5mM	8.19	0.38	7.78	0.68	9.09	0.99	12.41	7.50
1.75mM	8.68	3.17	8.07	0.98	8.07	0.56	6.27	2.30
1.25mM	8.23	1.10	8.07	0.60	9.35	2.53	8.11	0.56
0.9mM	7.07	1.35	8.02	1.39	7.64	0.93	6.60	0.50
0.625mM	7.73	2.33	7.91	0.98	8.08	1.32	7.49	1.01

Amino acid	Val		Pro		Phe		Tyr	
conc.	mean	SD	mean	SD	mean	SD	mean	SD
7mM	83.05	8.55	94.80	6.04	61.12	8.40	na	
5mM	77.43	19.59	72.30	3.52	50.83	3.88	na	
3.5mM	68.81	27.47	7.63	0.16	34.63	5.10	na	
2.5mM	11.58	4.16	8.77	1.02	8.80	2.46	na	
1.75mM	7.38	0.28	7.64	0.53	7.41	0.30	40.75	15.95
1.25mM	8.03	0.94	8.80	1.19	7.37	1.06	10.46	1.27
0.9mM	7.77	1.08	14.26	10.65	6.90	1.38	9.18	0.58
0.625mM	7.24	0.35	7.50	1.17	7.92	0.58	11.78	3.49

Amino acid	Trp		Ser		Thr		Met (I)	
conc.	mean	SD	mean	SD	mean	SD	mean	SD
7mM	55.80	14.15	85.58	9.05	81.79	19.80	94.14	10.23
5mM	57.33	3.77	62.31	18.31	49.59	12.77	95.27	17.31
3.5mM	89.23	20.73	34.12	10.63	23.14	4.30	80.54	16.13
2.5mM	111.42	9.77	8.08	0.44	7.06	0.69	77.44	27.60
1.75mM	84.77	21.26	7.50	0.96	7.62	0.68	61.57	33.26
1.25mM	81.29	6.68	7.87	0.73	8.14	0.26	7.62	0.99
0.9mM	82.79	22.72	7.47	0.89	7.16	0.77	7.29	0.55
0.625mM	35.86	44.10	7.53	0.88	7.03	0.63	8.87	1.06

Amino acid	Met (II)		Cys		Lys		Arg	
conc.	mean	SD	mean	SD	mean	SD	mean	SD
7mM	97.69	7.76	102.76	12.47	75.07	20.57	89.59	9.05
5mM	90.82	6.02	104.06	8.87	76.14	17.56	98.37	15.17
3.5mM	85.00	17.69	103.46	11.87	72.40	15.71	95.01	14.23
2.5mM	84.44	16.81	86.52	4.95	40.76	43.50	92.63	13.22
1.75mM	66.32	22.34	80.10	16.85	7.51	0.65	15.98	11.45
1.25mM	6.74	1.70	9.74	0.45	7.30	1.37	4.67	1.63
0.9mM	5.90	2.46	7.65	0.79	6.65	0.28	4.84	4.76
0.625mM	9.11	1.82	7.04	0.89	7.25	1.99	3.47	2.70

Amino acid	His		Asn		Gln		Asp	
conc.	mean	SD	mean	SD	mean	SD	mean	SD
7mM	94.86	27.20	105.88	26.45	89.38	12.49	61.65	27.51
5mM	24.67	16.81	87.02	9.06	73.18	9.79	52.11	23.90
3.5mM	20.41	10.54	63.56	15.56	72.30	8.66	50.84	32.15
2.5mM	25.13	19.11	29.60	23.67	17.09	18.49	11.75	3.80
1.75mM	7.64	1.31	7.99	0.13	7.45	0.73	6.14	4.81
1.25mM	10.94	3.29	8.07	1.47	7.89	0.41	8.32	0.42
0.9mM	7.38	0.58	7.33	0.71	8.50	0.64	8.09	1.32
0.625mM	7.06	0.45	7.16	0.48	15.06	11.38	8.84	1.28

Amino acid	Glu		water control (I)		water control (II)	
conc.	mean	SD	mean	SD	mean	SD
7mM	96.28	8.09	7.44	0.28	6.92	0.64
5mM	91.40	10.00	7.85	0.70	12.08	4.36
3.5mM	92.03	12.27	7.27	0.63	8.43	0.88
2.5mM	47.01	34.37	7.34	0.64	9.45	3.72
1.75mM	8.41	1.30	5.89	2.22	7.62	0.56
1.25mM	7.75	0.80	7.68	0.75	6.81	3.84
0.9mM	7.72	1.34	7.15	1.03	6.77	2.77
0.625mM	6.76	0.74	6.58	1.67	7.71	1.56

[a]Growth in all cases was determined in YEPD broth by OD_{600} readings after 24 h. Values shown are for % growth relative to control (no EW treatment). Mean values are shown from biological triplicates (except lysine, where n=2).

Table S3: Related to Figure S4. Inactivation of fungicidal EW activity by certain amino acids determined from subsequent growth for extended periods

Amino acid conc.	Arg, 24h		Arg, 48h		Arg, 72h	
	mean[a]	SD	mean	SD	mean	SD
7mM	89.59	9.05	96.11	0.35	96.33	0.86
5mM	98.37	15.17	99.88	2.21	99.76	2.35
3.5mM	95.01	14.23	96.74	3.37	97.30	1.93
2.5mM	92.63	13.22	94.35	3.67	95.84	1.75
1.75mM	15.98	11.45	53.24	32.31	95.61	3.44
1.25mM	4.67	1.63	8.55	14.11	32.44	55.60
0.9mM	4.84	4.76	0.49	0.49	0.40	0.41
0.625mM	3.47	2.70	0.30	0.29	0.28	0.22

Amino acid conc.	His, 24h		His, 48h		His, 72h	
	mean	SD	mean	SD	mean	SD
7mM	94.86	27.20	104.67	3.91	104.61	4.17
5mM	24.67	16.81	79.35	27.15	101.74	2.93
3.5mM	20.41	10.54	76.50	31.54	102.46	2.27
2.5mM	25.13	19.11	69.90	35.35	100.38	1.20
1.75mM	7.64	1.31	19.91	33.25	35.40	58.45
1.25mM	10.94	3.29	30.41	26.04	69.43	57.77
0.9mM	7.38	0.58	29.47	18.74	99.65	1.69
0.625mM	7.06	0.45	14.70	24.08	36.40	60.65

Amino acid conc.	Asn, 24h		Asn, 48h		Asn, 72h	
	mean	SD	mean	SD	mean	SD
7mM	105.88	26.45	104.26	9.29	102.59	5.52
5mM	87.02	9.06	105.04	1.63	103.90	2.73
3.5mM	63.56	15.56	88.06	12.39	97.20	4.28
2.5mM	29.60	23.67	37.05	21.67	94.40	4.89
1.75mM	7.99	0.13	10.07	4.48	93.28	9.69
1.25mM	8.07	1.47	6.52	3.51	92.28	2.94
0.9mM	7.33	0.71	6.60	5.02	70.85	50.70
0.625mM	7.16	0.48	4.25	6.06	34.10	57.99

Amino acid conc.	Met (II), 24h		Met (II), 48h		Met (II), 72h	
	mean	SD	mean	SD	mean	SD
7mM	97.69	7.76	102.71	4.35	100.71	5.16
5mM	90.82	6.02	104.84	2.94	103.23	2.20
3.5mM	85.00	17.69	90.86	12.64	97.43	1.97
2.5mM	84.44	16.81	88.72	14.10	99.54	1.58
1.75mM	66.32	22.34	87.05	26.35	101.00	6.66
1.25mM	6.74	1.70	16.60	12.22	91.89	11.64
0.9mM	5.90	2.46	22.30	25.98	65.90	57.07
0.625mM	9.11	1.82	10.71	17.30	34.26	58.25

Amino acid conc.	Ala, 24h		Ala, 48h		Ala, 72h	
	mean	SD	mean	SD	mean	SD
7mM	80.23	7.15	97.25	4.16	98.97	2.22
5mM	85.25	11.19	96.62	8.67	99.36	4.43
3.5mM	75.88	12.99	93.91	3.06	99.22	1.60
2.5mM	7.78	0.68	4.26	6.13	28.90	47.13
1.75mM	8.07	0.98	0.77	0.02	1.23	0.92
1.25mM	8.07	0.60	0.71	0.05	1.21	0.94
0.9mM	8.02	1.39	0.72	0.09	1.03	0.68
0.625mM	7.91	0.98	0.89	0.13	0.81	0.23

Amino acid conc.	water control (I+II)		water control (I+II)		water control (I+II)	
	mean	SD	mean	SD	mean	SD
7mM	7.18	0.42	15.81	16.44	52.12	50.60
5mM	9.96	1.85	19.22	17.46	51.08	48.71
3.5mM	7.85	0.75	9.27	9.72	33.12	28.11
2.5mM	8.39	1.78	13.16	21.57	17.45	28.92
1.75mM	6.76	1.12	2.20	2.79	17.85	30.05
1.25mM	7.24	1.55	0.65	0.21	0.68	0.32
0.9mM	6.96	1.18	3.39	4.48	16.21	26.90
0.625mM	7.14	1.31	0.76	0.12	0.60	0.12

[a]Growth in all cases was determined in YEPD broth by OD_{600} readings after 24 h, 48 h and 72 h. Values shown are for % growth relative to control (no EW treatment). Mean values are shown from biological triplicates (water control: mean value from the replicates [2 technical x 3 biological for each given mean value]).

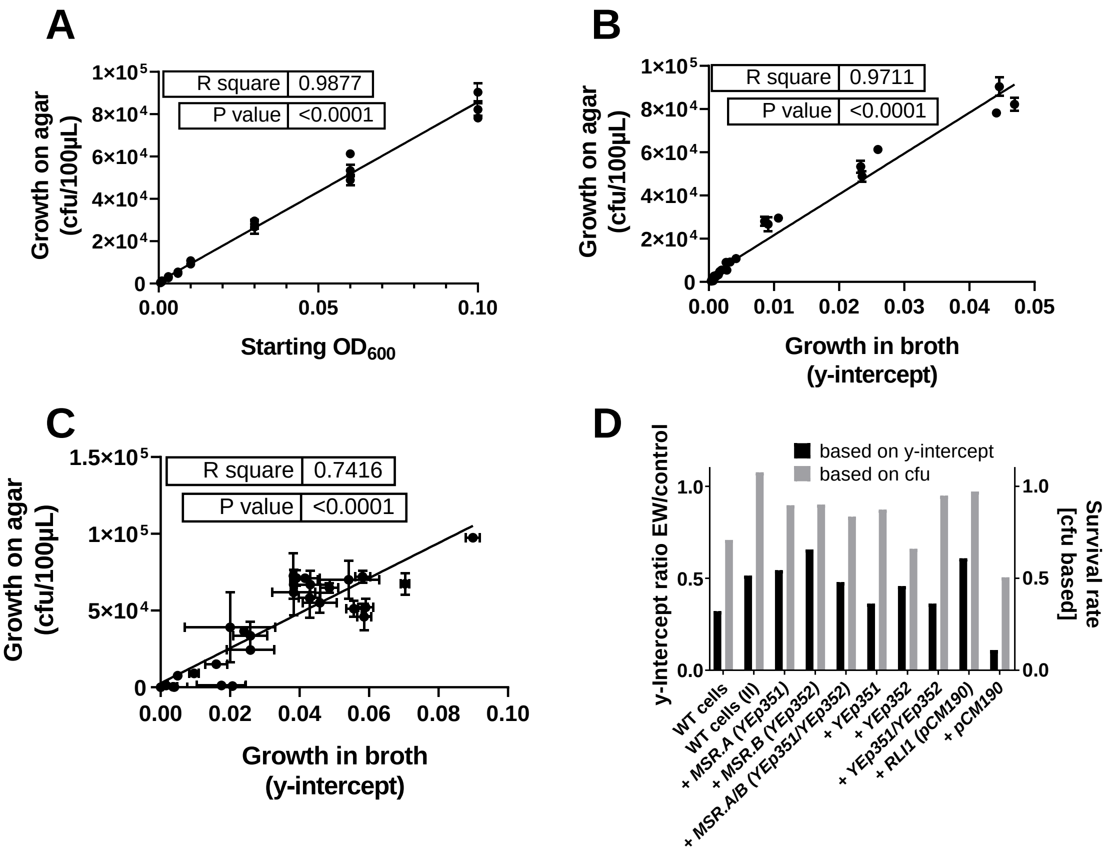

Figure S1: The y-intercept method for estimating survival in broth cultures. A,B,C) *S. cerevisiae* BY4741 was diluted to different starting densities (A,B) or treated with increasing concentrations of EW (C). Growth on agar was according to colony counts, determined by plating appropriate dilutions and enumeration of colony forming units (cfu), given as cfu per 100 µL undiluted suspension. For growth in broth, 100 µL cells were cultured in 96 well plates in a BioTek® microplate reader (30°C, 1096 cycles min^{-1}) and, for the period of highest growth rate (maximum slope in an exponential regression), the y-intercept was determined (see Methods). Following EW treatment (C), the correlation between cfu (agar) and y-intercept (broth) was a little weaker than with non-stressed cell suspensions (A) (R^2=0.74 vs. R^2=0.97). Data are from at least three biological replicates, individual data points are shown ± SD of technical replicates. D) *S. cerevisiae* BY4741 transformed or not with the indicated plasmids were treated with EW (0.5 mg L^{-1} FAC) for 5 min before survival was assessed on agar and in broth as described for A-C. WT, wild type. Survival of EW treatment calculated from the y-intercept was generally lower than that based on cfu but very similar relative effects of the genetic manipulations could be observed with both methods, e.g., higher survival of the *RLI1* overexpressing strain compared to its empty vector control. Data are averaged from two technical replicates. The results support use of growth extrapolation to the y-intercept for estimating starting viable-cell density. The y-intercept results may be affected by non-killing effects such as growth delays following stress (Lu et al., 2009), which cannot be easily detected by colony enumeration. To reflect this, y-intercept based rates are termed recovery rates throughout.

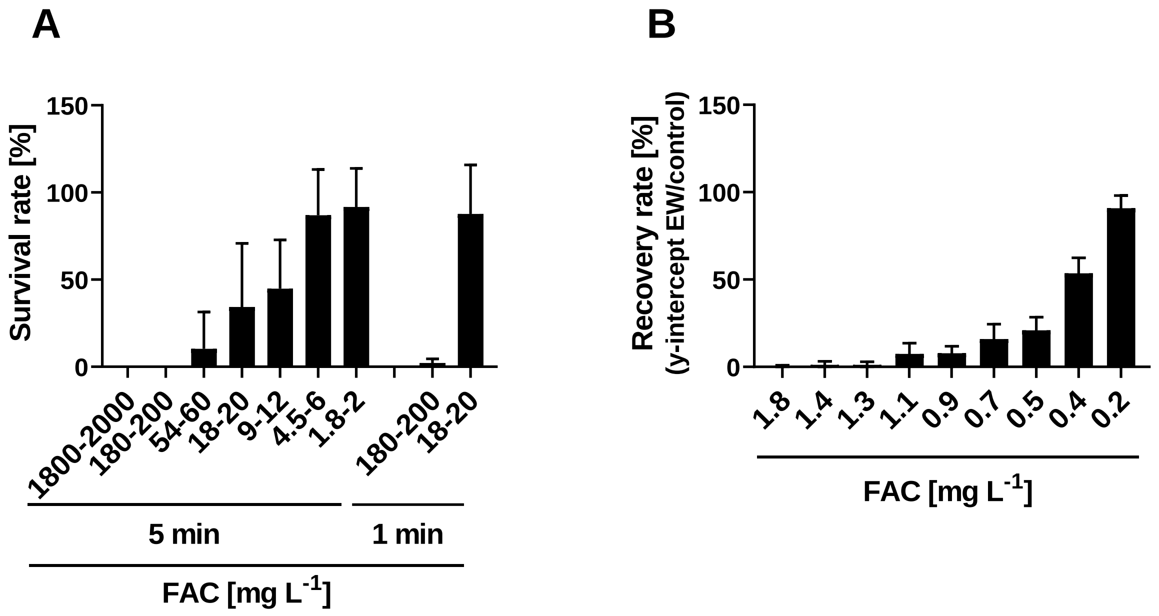

Figure S2: Dose-dependent survival of fungi after EW treatment. *A. niger* spores (A) or vegetative *S. cerevisiae* BY4741 cells (B) were mixed with EW at the indicated FAC concentrations and incubated for 5 min except where 1 min is specified. A) Survival rates were determined by colony counts on YEPD agar. B) Survival of yeasts after treatment was estimated by subsequent recovery in YEPD broth (see Methods and Figure S1). Values shown are means from at least 3 biological replicates ± SD.

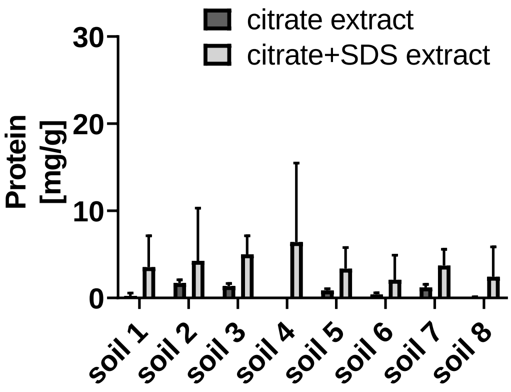

Figure S3: Protein determination in soil extracts. Organic compounds from soil samples (characteristics listed in Table S1) were extracted with protocols using either citrate or citrate + SDS (see Materials and Methods) and the protein content measured with a modified Lowry assay (Redmile-Gordon et al., 2013). Values are means of two technical replicates ± SD. No protein was detected in at least one of the technical replicates for soils 1, 4 and 8 (with both extraction methods) and soil 2 (citrate + SDS extract).

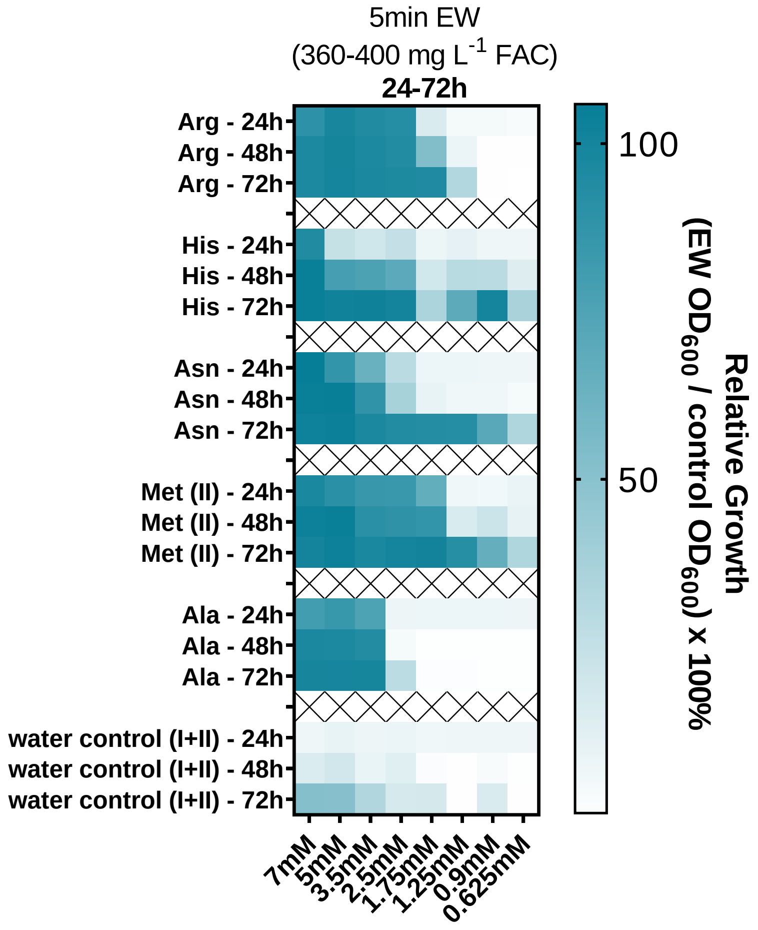

Figure S4: Related to Figure 2E. Inactivation of fungicidal EW activity by certain amino acids becomes apparent at later time points. Amino acids were included at the indicated concentrations in EW preparations (360-400 mg L^{-1} FAC) 5 min prior to 5-min EW treatment of *A. niger* spores. Growth was determined in YEPD broth by OD_{600} readings after 24 h, 48 h and 72 h and normalised to control growth without EW treatment. Mean values are shown from biological triplicates. Numerical values and standard deviations are listed in Table S3.

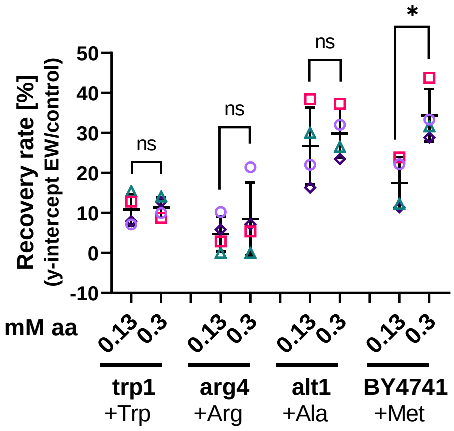

Figure S5: Influence of cellular amino acids on recovery of auxotrophic yeast strains after EW treatment. Survival of yeasts after EW treatment (0.5-1 mg L^{-1} FAC, 5 min) was estimated by subsequent recovery in YEPD broth (see Methods and Figure S1). *Saccharomyces cerevisiae* BY4741 (a *Δmet15* methionine auxotroph) and isogenic deletion strains (*Δtrp1,* tryptophan auxotroph; *Δarg4,* arginine auxotroph; *Δalt1,* partial alanine auxotroph) were pre-cultured with their required amino acid either at 0.13 mM or at 0.3 mM total concentration, as indicated, for 4–5 h prior to EW treatment. The growth medium was YNB broth (buffered, 0.1 M potassium phosphate buffer pH 6) which also routinely included 0.1338 mM Met, 0.129 mM His, 0.763 mM Leu, 0.178 mM Ura. For Met, 0.3 mM refers to the total final concentration in YNB (i.e. including the 0.13 mM), as opposed to supplementation only. Cells were washed in water before EW treatment. Mean values ± SD are shown for at least three biological replicates, with different replicate experiments distinguished by different symbols. *$p<0.05$; ns, not significant; according to paired *t*-test (two-tailed) with correction for multiple comparisons by controlling the false discovery rate at 5% FDR (Benjamini et al., 2006).

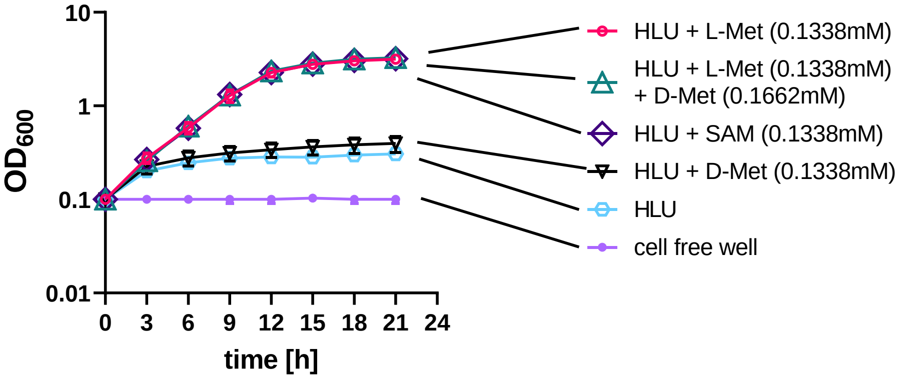

Figure S6: Yeast growth in minimal medium supplemented with different Met sources. Culture density of *S. cerevisiae* BY4741 was determined at 3 h intervals in a shaking plate reader (starting OD_{600}=0.1). The growth medium was YNB broth (buffered, 0.1 M phosphate buffer pH 6) supplemented with 0.129 mM His, 0.763 mM Leu, 0.178 mM Ura (HLU) plus Met or S-adenosyl-methionine (SAM) at the indicated concentrations. Mean values ± SD are shown for three biological replicates.

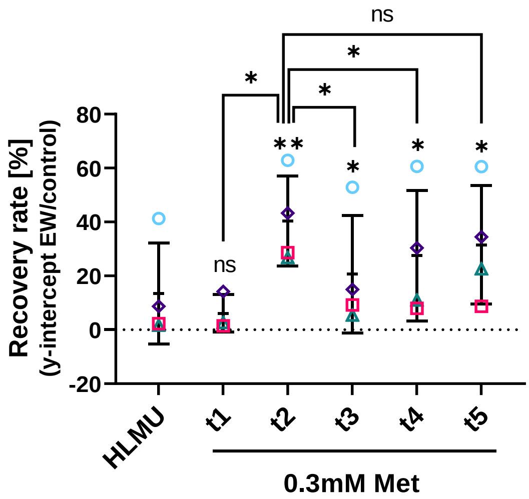

Figure S7: Influence of pre-culture with methionine for varying durations on recovery of cells after EW treatment. Survival of yeasts after EW treatment (0.5-1 mg L^{-1} FAC, 5 min) was estimated by subsequent recovery in YEPD broth (see Methods and Figure S1). Pre-culture of *S. cerevisiae* BY4741 with methionine at the indicated concentration for varying durations during exponential growth: t1, <0.5 min; t2, 10 min; t3, 2–2.5 h; t4, 4–5 h; t5, 20 h (with an intervening sub-culture after 16 h). Control growth (HLMU) was in YNB broth containing 0.1338 mM Met, 0.129 mM His, 0.763 mM Leu, 0.178 mM Ura, and Met was added to reach a total concentration of 0.3 mM for pre-culture conditions. Cells were washed in water before EW treatment. Mean values ± SD are shown for at least three biological replicates, with different replicate experiments distinguished by different symbols. *p<0.05, **p<0.01, ns, not significant; according to paired *t*-test (two-tailed), comparing between HLMU control and Met pre-culture unless shown by a connecting line, with correction for multiple comparisons by controlling the false discovery rate at 5% FDR (Benjamini et al., 2006).

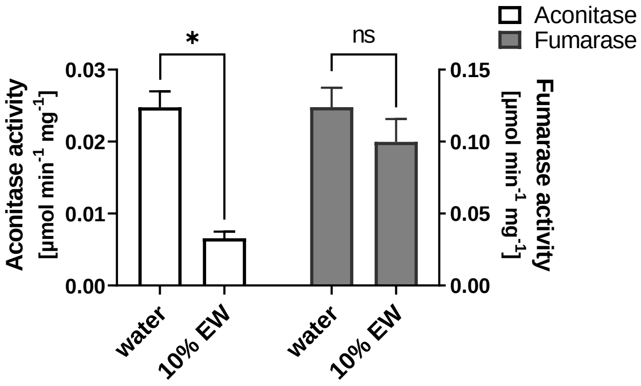

Figure S8: Related to Figure 7A. Aconitase and fumarase activities following *in vitro* EW treatment. Crude protein extracts from exponentially growing yeast cells were treated *in vitro* with 10% EW [v/v] (180-200 mg L^{-1} FAC) or water (control) for 20 min before determination of aconitase and fumarase activities. Mean values ± SD are shown for three biological replicates. *$p<0.05$, ns, not significant; according to paired *t*-test (two-tailed), with correction for multiple comparisons by controlling the false discovery rate at 5% FDR (Benjamini et al., 2006).
